# Pilates-Based Exercise and Its Impact on Nutritional Status and Health-Related Quality of Life in Older Adults with Type 2 Diabetes: A Randomized Controlled Trial

**DOI:** 10.3390/diagnostics15222913

**Published:** 2025-11-18

**Authors:** Beatriz Ruiz-Ariza, Agustín Aibar-Almazán, Fidel Hita-Contreras, Yolanda Castellote-Caballero, María del Carmen Carcelén-Fraile

**Affiliations:** 1 Department of Health Sciences, Faculty of Health Sciences, University of Jaén, 23007 Jaén, Spainmycastel@ujaen.es (Y.C.-C.); 2 Department of Educational Sciences, Faculty of Social Sciences, University of Atlántico Medio, 35017 Las Palmas de Gran Canaria, Spain

**Keywords:** type 2 diabetes mellitus, Pilates, nutritional status, health-related quality of life, randomized controlled trial

## Abstract

**Background/Objectives**: Type 2 diabetes mellitus (T2DM) is a prevalent chronic disease frequently associated with impaired nutritional status and reduced health-related quality of life (HRQoL), especially in older adults. Alongside pharmacological treatment and diet, physical exercise has emerged as a complementary strategy. Pilates, a mind–body discipline focused on controlled movement and postural alignment, may help improve outcomes beyond conventional care. This study aimed to evaluate the effectiveness of a 12-week Pilates intervention on nutritional status and HRQoL in older adults with T2DM. **Methods**: A randomized controlled trial was conducted with 104 older adults diagnosed with T2DM. The participants were randomly allocated to a Pilates group (*n* = 52) or a control group (*n* = 52). The intervention consisted of 24 supervised sessions delivered twice weekly for 60 min over 12 weeks. Nutritional status was assessed using the Mini Nutritional Assessment (MNA), and HRQoL was measured with the 12-Item Short Form Health Survey (SF-12). Both assessments were carried out at baseline and after the intervention. **Results**: Post-intervention scores indicated better nutritional condition and higher ratings in both physical and mental dimensions of HRQoL, while no significant changes were observed in controls. **Conclusions**: A structured Pilates program improved nutritional status and HRQoL in older adults with T2DM. These results suggest that Pilates is a feasible, safe, and effective complementary therapy in the comprehensive management of this population.

## 1. Introduction

Type 2 diabetes mellitus (T2DM) is a chronic metabolic disorder characterized by persistent hyperglycemia due to insulin resistance, impaired insulin secretion, or both [1]. It represents one of the major public health challenges of the 21st century. According to the International Diabetes Federation, the prevalence of diabetes has risen dramatically in the last four decades, from 108 million adults in 1980 to 537 million in 2021, and projections estimate an increase to 783 million by 2045 [2]. The disease is associated with severe complications, including cardiovascular disease, nephropathy, neuropathy, and retinopathy, which contribute to disability and premature mortality [3]. Beyond its clinical burden, T2DM places an enormous strain on healthcare systems worldwide, both in direct costs and in loss of productivity.

Older adults are disproportionately affected by T2DM, partly due to age-related physiological changes that exacerbate the progression and consequences of the disease. Aging is accompanied by sarcopenia, decreased balance, and functional limitations, which can interact with the complications of diabetes to increase frailty and dependency [4]. At the same time, the psychological and social changes associated with aging may further worsen disease management and overall well-being [5]. Two particularly relevant aspects in this context are health-related quality of life (HRQoL) and nutritional status. Malnutrition is common in older adults with T2DM and has been linked to increased morbidity, higher risk of hospitalization, and mortality [6]. On the other hand, HRQoL captures the multidimensional impact of diabetes on physical, psychological, and social functioning, and is considered a key outcome in chronic disease management [7].

Lifestyle interventions are recognized as central components in the prevention and treatment of T2DM. Alongside pharmacological therapy, maintaining a balanced diet and engaging in regular physical activity are strongly recommended [8]. Exercise contributes to glycemic control, weight management, cardiovascular health, and psychological well-being [9]. International guidelines, including those jointly issued by the American Diabetes Association (ADA) and the American College of Sports Medicine (ACSM), advocate a combination of aerobic and resistance exercise as the most effective approach for individuals with diabetes [10,11]. However, despite this evidence, adherence to exercise programs remains low, especially in older populations where comorbidities, functional limitations, and motivational barriers play an important role [12]. This highlights the need for exercise modalities that are safe, adaptable, and enjoyable to ensure long-term adherence.

Pilates is a mind–body exercise method created in the early 20th century by Joseph Pilates. It emphasizes controlled movement, breathing, concentration, and postural alignment, aiming to improve core strength, flexibility, balance, and overall body awareness [13]. Unlike high-intensity activities, Pilates is low-impact and can be adapted to different levels of physical ability, making it particularly suitable for older adults [14]. Beyond its physical benefits, Pilates also integrates a mental component that promotes relaxation, stress reduction, and greater connection between body and mind [15].

In recent years, Pilates has gained growing attention in the scientific literature. Studies conducted in older populations have reported improvements in strength, flexibility, balance, and postural stability [16,17]. Moreover, evidence suggests that Pilates may have positive effects on psychosocial outcomes, including reductions in anxiety and depressive symptoms, as well as improvements in self-perceived health and quality of life [18,19]. Some research has also indicated potential benefits for individuals with chronic conditions, such as musculoskeletal disorders, obesity, or diabetes, although findings remain heterogeneous [20].

Specifically in people with T2DM, mind–body exercises such as yoga, tai chi, or Pilates have been investigated as complementary interventions to conventional care. Multiple studies have shown enhancements in glycemic regulation, body composition, and HRQoL [21,22,23]. However, results across studies are inconsistent, largely due to variations in study design, intervention duration, frequency, and outcome measures [24]. In some cases, Pilates interventions have been combined with other modalities, making it difficult to isolate their specific effects. Furthermore, while there is a growing body of research on functional and psychological outcomes, few studies have directly addressed its impact on nutritional status, despite the clinical relevance of this factor in older adults with diabetes [25].

The association between exercise and improved nutritional status has been hypothesized through several mechanisms. Physical activity may increase appetite regulation, promote better food choices, and reduce sarcopenia, thereby contributing to better overall nutritional profiles [26]. In older adults with T2DM, these mechanisms could be particularly valuable, since both malnutrition and excess adiposity frequently coexist and complicate disease management [27]. However, empirical evidence specifically linking Pilates to changes in nutritional status remains scarce, which underlines the importance of further research in this area.

Another key dimension is HRQoL, which integrates physical health, mental well-being, and social participation. In patients with T2DM, HRQoL is often lower compared to the general population, reflecting both the direct burden of symptoms and complications, as well as the psychosocial impact of long-term treatment regimens [28]. Improving HRQoL is increasingly recognized as a central goal in diabetes management, complementing traditional biomedical outcomes such as glycemic control [29]. Although some studies suggest that Pilates can improve HRQoL, evidence remains inconclusive, and further trials are needed to confirm these benefits in populations with chronic disease [30].

Given the rising prevalence of T2DM, the vulnerabilities of older adults, and the importance of addressing nutritional status and HRQoL, there is a strong rationale for exploring Pilates as a complementary therapy. Pilates offers a safe, adaptable, and potentially enjoyable form of physical activity that could contribute to the multidimensional management of diabetes. Nevertheless, the current literature is insufficient to establish definitive conclusions regarding its impact on these outcomes [24,31].

The main objective of this randomized controlled trial was to evaluate the effectiveness of a 12-week Pilates-based exercise program in improving nutritional status in older adults with T2DM. The secondary objective was to assess the program’s impact on HRQoL, including both physical and mental health domains. We hypothesized that participants in the Pilates group would experience significant improvements in both nutritional status and HRQoL compared with controls.

## 2. Materials and Methods

### 2.1. Study Design and Participants

This investigation was structured as a randomized controlled clinical trial with two parallel arms (Pilates-based intervention vs. control group), designed to evaluate the effects of a structured exercise program on older adults with type 2 diabetes mellitus (T2DM). Before commencing the intervention phase, all potential participants were fully informed about the objectives, procedures, possible risks, and expected benefits of the study through a detailed information sheet. Informed consent was obtained in writing from each participant, guaranteeing voluntary participation and the right to withdraw at any stage without consequences for their standard care. Ethical clearance was obtained from the University of Jaén’s Ethics Committee (FEB.23/3.TES). All phases of the trial were implemented in strict accordance with the ethical principles established by the World Medical Association in the Declaration of Helsinki, which governs the protection of human subjects in biomedical research. Particular attention was paid to safeguarding confidentiality, data protection, and the safety of participants throughout the study. Furthermore, the clinical trial was prospectively registered at ClinicalTrials.gov, where it was assigned the registration number NCT05711602. This ensured transparency of the research protocol, facilitated international visibility, and enabled comparison with similar interventions conducted in other populations.

### 2.2. Recruitment and Sampling

**Figure 1:**
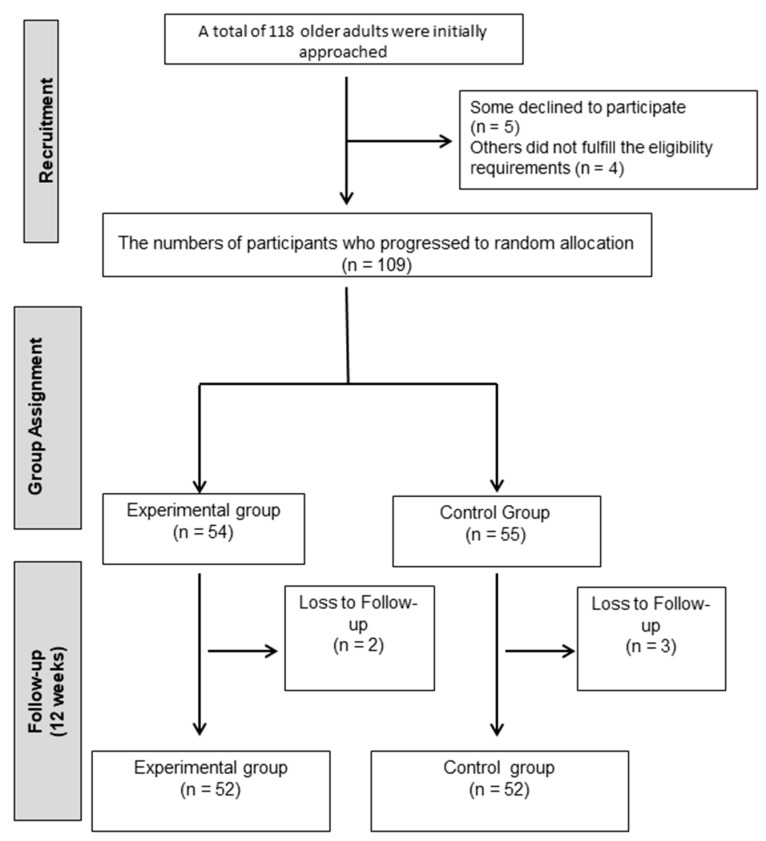
Participant flowchart.

Older adults from Jaén with a confirmed diagnosis of type 2 diabetes mellitus (T2DM) were invited to participate. Recruitment was carried out through local healthcare services, where clinicians identified potential candidates and verified eligibility according to predefined criteria. The inclusion criteria required participants to: (i) have a medical diagnosis of T2DM; (ii) be 65 years of age or older; (iii) present sufficient functional capacity to perform the planned Pilates exercises safely; (iv) not be engaged in any structured exercise program during the recruitment phase; and (v) have the capacity to undergo all clinical assessments and fill out self-reported questionnaires. The exclusion criteria were: (i) presence of systemic conditions affecting mobility or physical performance (e.g., severe musculoskeletal, neurological, or ophthalmological disorders); (ii) diagnosis of vestibular dysfunction; or (iii) regular intake of pharmacological treatments that could alter balance or coordination, such as sedatives, antidepressants, or anxiolytics. Of the 118 individuals initially screened, 5 declined to participate and 4 did not meet the eligibility criteria. The final sample comprised 109 participants, who were randomly assigned to either the Pilates intervention group or the control group. Figure 1 provides a flow diagram of the selection process.

### 2.3. Randomization

In order to guarantee methodological rigor and reduce the risk of bias, participants were randomized after baseline assessment. The allocation sequence was generated using a computerized list of random numbers prepared by an independent researcher who was not involved in recruitment, evaluation, or delivery of the intervention. Group assignments were placed in sealed, opaque and identical envelopes (same size, color and appearance) to ensure concealment until the moment of allocation. Blinding procedures were also applied. Outcome assessments were performed by an evaluator who remained unaware of participants’ group assignments throughout the study. This approach helped reduce the risk of measurement bias and reinforced the internal validity of the trial. Following randomization, participants were distributed into two arms: the experimental group (Pilates-based exercise program) and the control group. Ultimately, 55 individuals were randomly to the intervention group and 54 to the control group. Those allocated to the control condition were instructed to continue with their habitual daily routines and to refrain from participating in other structured physical exercise programs during the study period. As part of good clinical practice, all participants, including controls, received general recommendations encouraging safe physical activity adapted to their health status. In addition, participants allocated to the control group were instructed to maintain their usual daily routines and to refrain from initiating any structured physical exercise programs during the study period. Compliance was monitored through weekly telephone follow-ups to ensure that no additional exercise interventions were undertaken.

### 2.4. Sample Size Calculation

The sample size was calculated using G*Power (version 3.1.9.7) for a two-group pre–post design, assuming a significance level of α = 0.05, a statistical power of 0.80, and an expected moderate effect size (Cohen’s d = 0.44) for the main outcomes (nutritional status and health-related quality of life). Under these assumptions, a total of 104 participants (52 per group) was sufficient to detect meaningful differences between the intervention and control groups. This number was set as the final sample to ensure adequate statistical power while maintaining feasibility in recruitment.

### 2.5. Outcomes

The assessment of study outcomes was conducted by an independent researcher blinded to the intervention. Data were collected at two points, prior to group allocation and immediately after the intervention period. Sociodemographic variables included age, sex, years since diabetes diagnosis, comorbidities, family history of diabetes, tobacco use and alcohol use.

#### 2.5.1. Nutritional Status

Nutritional status was evaluated using the Mini Nutritional Assessment (MNA), a validated screening tool designed for older adults [32,33]. The MNA includes questions on anthropometric measures, dietary intake, general health, and self-perception of nutritional and health status. The total score ranges from 0 to 30, with higher values indicating better nutritional status. According to standardized cut-off points, scores ≥24 indicate normal nutritional status, 17–23.5 suggest risk of malnutrition, and <17 denote malnutrition. This instrument has been widely used in clinical and research settings for detecting malnutrition in elderly populations.

#### 2.5.2. Health-Related Quality of Life (HRQoL)

The 12-Item Short Form Health Survey (SF-12) was employed to measure HRQoL [34]. This questionnaire evaluates two main components: the Physical Component Summary (PCS) and the Mental Component Summary (MCS), which together provide a multidimensional perspective of self-perceived health status. Scores are standardized to a mean of 50 with a standard deviation of 10 in the general population, with higher scores reflecting better health-related quality of life. The SF-12 has demonstrated good reliability and validity in both clinical and general populations, including individuals with chronic conditions such as T2DM.

### 2.6. Intervention

**Figure 2:**
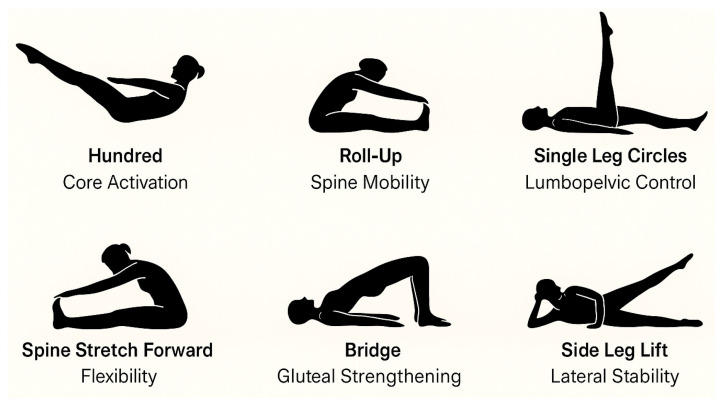
Main Pilates Postures and Exercises Performed.

The intervention consisted of a 12-week Pilates-based exercise program with a total of 24 sessions, delivered twice weekly for 60 min each. The program was conducted at the therapeutic exercise room of a primary healthcare center, equipped with mats, elastic bands, and Pilates circles. Sessions were delivered in small groups of 8–10 participants, each supervised by a certified physiotherapist trained in clinical Pilates and assisted by a research staff member to ensure safety and adherence. All sessions were conducted in small groups by a certified Pilates instructor with over 450 h of specialized training in therapeutic applications for older adults. The program was structured into three phases to ensure safety, gradual progression, and comprehensive physical conditioning. The initial warm-up phase (≈10 min) focused on breathing control (e.g., lateral thoracic breathing), postural awareness in standing and seated positions, and global joint mobilization, including cervical circles, shoulder rolls, hip rotations, and gentle spinal articulation. The main phase (≈35 min) emphasized core activation and functional strengthening. Exercises were adapted to the participants’ physical capacities and progressed weekly in terms of difficulty (Figure 2).

**Figure 3:**
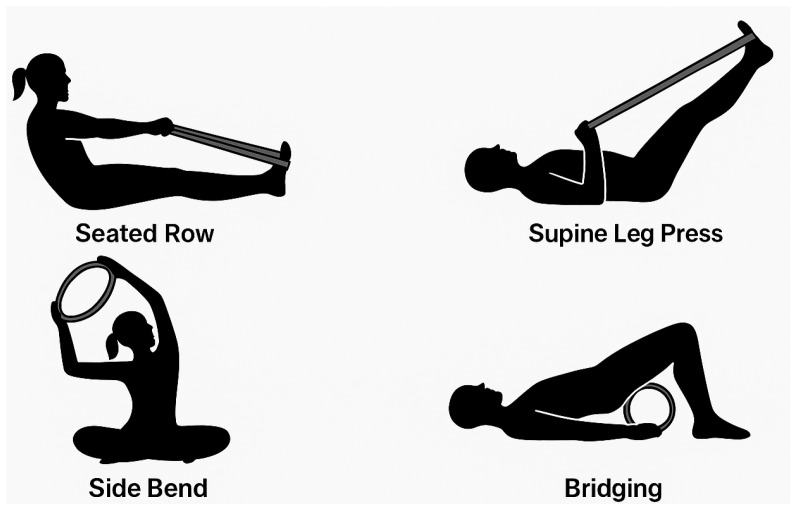
Main Pilates Postures and Exercises Using Elastic Bands or Pilates Circles.

Progression was achieved by gradually increasing repetitions (from 6 to 12), modifying the base of support (e.g., from bilateral to unilateral stance), introducing resistance with elastic bands or Pilates circles, and adding multiplanar movement patterns (Figure 3).

Safety and proper execution were prioritized, and feedback (both verbal and manual) was continuously provided to avoid compensatory patterns. In accordance with the mind–body principles of the Pilates method, participants were encouraged to maintain conscious control over their movements, focusing on breathing synchronization, precision, and body awareness throughout the sessions. Each exercise was accompanied by verbal cues promoting concentration, controlled breathing, and mindfulness of posture and muscle activation. Participants were instructed to consciously engage the “powerhouse” (core muscles) while maintaining mental focus on smooth, controlled execution. The final cool-down phase (≈15 min) included global static stretching of the main muscle groups (hamstrings, hip flexors, and pectorals), relaxation techniques, diaphragmatic, body awareness exercises and mindful breathing to further enhance mental relaxation, performed in a seated or supine position.

To ensure adherence and maximize engagement, several strategies were applied: participants received reminders via phone calls prior to sessions, instructors offered motivational feedback, and a supportive group environment was promoted to enhance social interaction. Attendance was systematically monitored through logs, and participants who faced difficulties received individualized encouragement. Adherence to the program was high, with an overall attendance rate of approximately 87%, and most participants completed at least 20 of the 24 sessions. Throughout the program, participants were instructed to maintain their regular medical treatment and lifestyle habits, ensuring that the observed outcomes could be attributed to the exercise intervention. The intervention sessions were monitored by certified staff, providing safety and methodological consistency.

### 2.7. Statistical Analysis

Data analysis was performed using SPSS software, version 20.0 (IBM Corp., Armonk, NY, USA). Statistical significance was set at *p* < 0.05. Descriptive statistics are expressed as mean and standard deviation (SD) for continuous variables, and as absolute frequencies and percentages for categorical variables. The normality of continuous variables was assessed using the Kolmogorov–Smirnov test prior to applying parametric analyses. Baseline differences between the experimental group (Pilates) and the control group were examined using the independent-samples Student’s *t*-test for continuous data and the chi-square test for categorical variables. Intervention effects were tested through a two-way mixed-design ANOVA, including group (intervention vs. control) as the between-subjects factor and time (pre- vs. post-intervention) as the within-subjects factor. When significant main or interaction effects were detected, Bonferroni-adjusted post hoc comparisons were performed to identify specific differences between groups and across time points. For all ANOVA models, effect sizes were estimated using partial eta squared (η^2^), with values of 0.01, 0.06 and 0.14 interpreted as small, medium and large effects, respectively. In addition, Cohen’s d was calculated for within-group (pre–post) and between-group comparisons, and interpreted as negligible (<0.20), small (0.20–0.49), moderate (0.50–0.79), or large (≥0.80).

## 3. Results

A total of 29.81% of the sample were men and 70.19% were women, with a mean age of 69.70 ± 6.44 years. The majority of participants were retired (57.26%), married (59.83%), and had attained primary education as their highest level of schooling (64.10%) (Table 1). No significant differences were found between groups in terms of sociodemographic characteristics.

**Figure 4:**
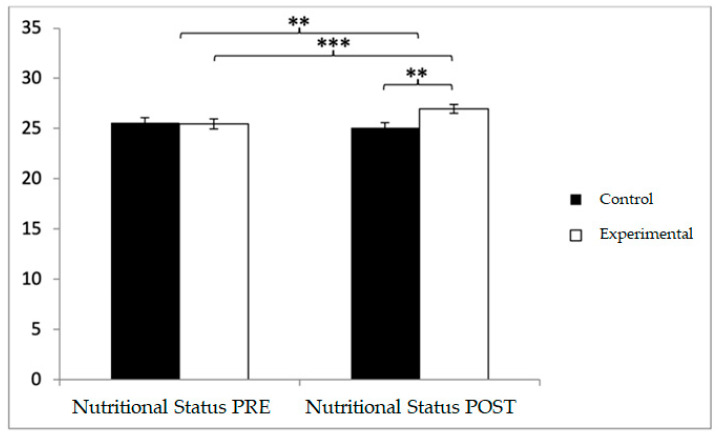
Inter- and intra-group comparisons for nutritional status. Bars represent mean ± SD. Horizontal lines indicate significant differences between groups or time points (*p* < 0.05). ** *p* < 0.01, *** *p* < 0.001.

With respect to nutritional status, participants in the control group (CG) reported higher baseline values (25.56 ± 3.73) compared to those in the experimental group (EG) (25.46 ± 3.50). However, in the post-intervention assessment, higher values were observed in the EG relative to the CG (26.98 ± 3.21 vs. 25.08 ± 3.59). Significant effects were found for Group × Time: F(1,102) = 42.102, *p* < 0.001, η^2^ = 0.292; Time: F(1,102) = 11.351, *p* = 0.001, η^2^ = 0.100; and Group: F(1,102) = 1.814, *p* = 0.017, η^2^ = 0.017 (Figure 4). Post hoc analysis revealed statistically significant differences between groups at the post-intervention measurement, t(102) = −2.853, *p* = 0.005, with a medium effect size (d = 0.56). Additionally, within-group analysis showed significant pre–post improvements in the EG, t(51) = −7.945, *p* < 0.001, with a small effect size (d = 0.45).

**Figure 5:**
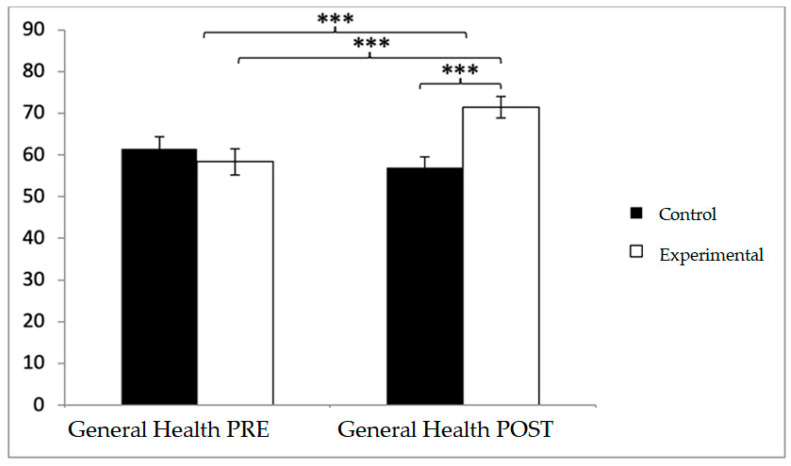
Inter- and intra-group comparisons for the general health domain of the SF-12. Bars represent mean ± SD. Horizontal lines indicate significant differences between groups or time points (*p* < 0.05). SF-12: Medical Outcome Study Short Form-12. *** *p* < 0.001.

For health-related quality of life, baseline differences favored the control group in several domains, but this tendency shifted after the intervention. In general health, participants in the control group reported higher values at baseline (61.54 ± 19.74 vs. 58.37 ± 22.49), whereas post-intervention scores were greater in the experimental group (71.44 ± 18.29 vs. 57.02 ± 18.37). Significant effects emerged for Group × Time (F(1,102) = 63.551, *p* < 0.001, η^2^ = 0.384) and Time (F(1,102) = 15.031, *p* < 0.001, η^2^ = 0.128). Post hoc tests showed significant between-group differences at post-intervention (t(1,102) = −4.012, *p* < 0.001; d = 0.79) and pre–post improvements within the experimental group (t(51) = −8.073, *p* < 0.001; d = 0.64) (Figure 5).

**Figure 6:**
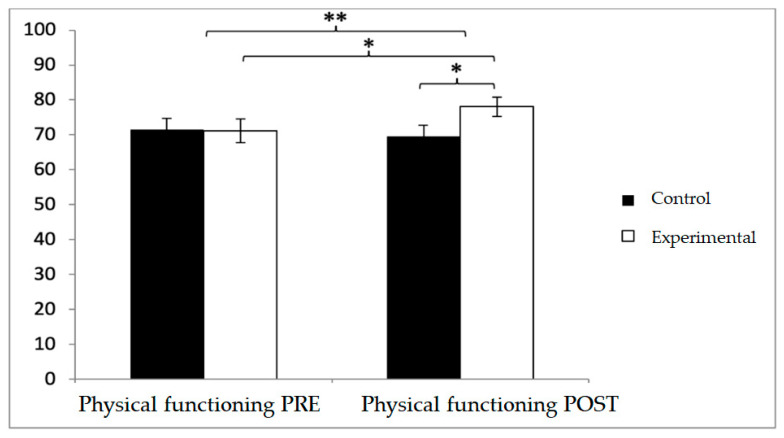
Inter- and intra-group comparisons for the physical functioning domain of the SF-12. Bars represent mean ± SD. Horizontal lines indicate significant differences between groups or time points (*p* < 0.05) * *p* < 0.05, ** *p* < 0.01.

A similar pattern was observed in physical functioning. While baseline values were higher in the control group (71.44 ± 23.71 vs. 71.15 ± 24.02), post-intervention values favored the experimental group (78.08 ± 19.83 vs. 69.52 ± 22.69). Significant effects were found for Group × Time (F(1,102) = 20.217, *p* < 0.001, η^2^ = 0.165) and Time (F(1,102) = 6.459, *p* = 0.013, η^2^ = 0.060). Between-group differences were significant at post-intervention (t(1,102) = −2.048, *p* = 0.043; d = 0.40), and within the experimental group, improvements were also detected (t(51) = −2.145, *p* = 0.037), although with a negligible effect size (d = 0.07) (Figure 6).

**Figure 7:**
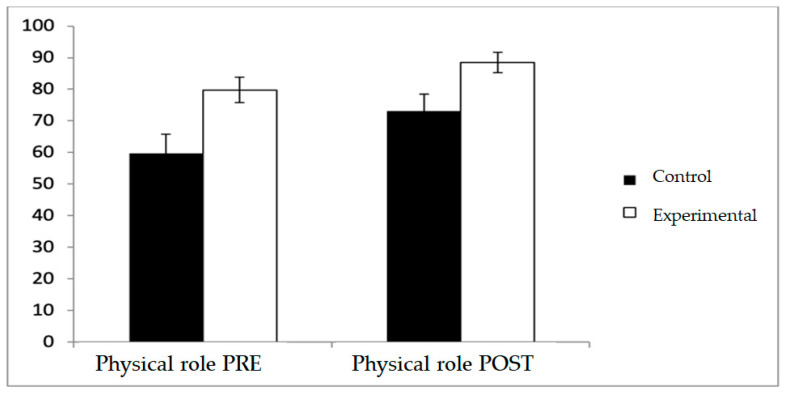
Inter- and intra-group comparisons for the physical role domain of the SF-12.

Regarding physical role, the experimental group consistently reported higher scores both before and after the intervention (79.81 ± 28.46 vs. 59.62 ± 44.32 at baseline; 88.46 ± 23.46 vs. 73.08 ± 38.93 at post-intervention) with a significant main effect for Group (F(1,102) = 9.335, *p* = 0.003, η^2^ = 0.084), whereas pre–post changes within each group did not reach statistical significance, and no Group × Time interaction was observed (Figure 7).

**Figure 8:**
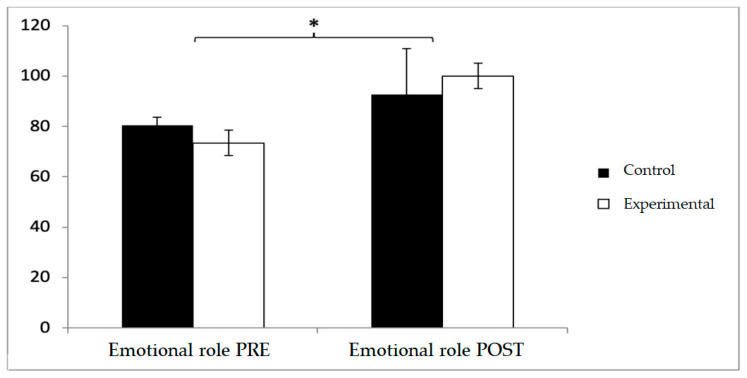
Inter- and intra-group comparisons for the emotional role domain of the SF-12. * *p* < 0.05. Bars represent mean ± SD. Horizontal lines indicate significant differences on time points (*p* < 0.05).

In emotional role, baseline values were higher in the control group (80.48 ± 22.47 vs. 73.46 ± 28.44), but post-intervention, the experimental group surpassed the control group (100 ± 32.21 vs. 92.67 ± 30.99). No significant effects were found for Group × Time or Group, although a significant effect was observed for Time (F(1,102) = 4.280, *p* = 0.041, η^2^ = 0.040) (Figure 8).

**Figure 9:**
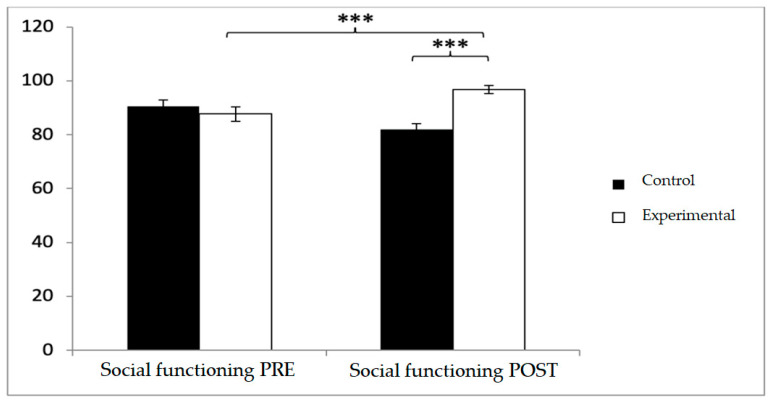
Inter- and intra-group comparisons for the social functioning domain of the SF-12. Bars represent mean ± SD. Horizontal lines indicate significant differences between groups or time points (*p* < 0.05). *** *p* < 0.001.

In social functioning, participants in the control group reported higher baseline scores (90.46 ± 17.19 vs. 87.65 ± 19.67), but post-intervention, the experimental group reported significantly higher values (96.69 ± 10.71 vs. 82.46 ± 14.82; *p* < 0.001). Significant effects were observed for Group × Time (F(1,102) = 29.003, *p* < 0.001, η^2^ = 0.221) and Group (F(1,102) = 4.486, *p* = 0.037, η^2^ = 0.042). Post hoc comparisons indicated significant between-group differences at post-intervention (t(1,102) = −5.613, *p* < 0.001; d = 1.10) and pre–post improvements in the experimental group (t(51) = −6.729, *p* < 0.001; d = 0.30), while no significant changes were detected in the control group (t(51) = 1.485, *p* > 0.05) (Figure 9).

**Figure 10:**
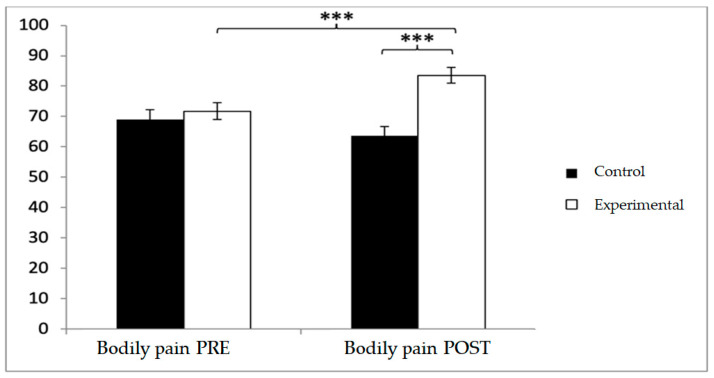
Inter- and intra-group comparisons for the bodily pain domain of the SF-12. Bars represent mean ± SD. Horizontal lines indicate significant differences between groups or time points (*p* < 0.05). *** *p* < 0.001.

For bodily pain, the experimental group scored higher at both baseline (71.75 ± 19.93 vs. 69.25 ± 22.90) and post-intervention (83.56 ± 18.28 vs. 63.56 ± 23.07). Significant effects were observed for Group × Time (F(1,102) = 24.210, *p* < 0.001, η^2^ = 0.192) and Group (F(1,102) = 24.210, *p* < 0.001, η^2^ = 0.192). Post hoc analyses confirmed significant between-group differences at post-intervention (t(1,102) = 6.937, *p* < 0.001; d = 0.96), as well as pre–post improvements within the experimental group (t(51) = −7.418, *p* < 0.001; d = 0.62) (Figure 10).

**Figure 11:**
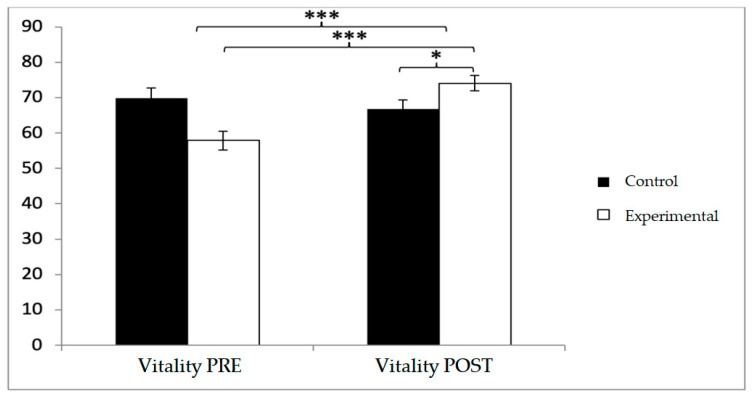
Inter- and intra-group comparisons for the vitality domain of the SF-12. Bars represent mean ± SD. Horizontal lines indicate significant differences between groups or time points (*p* < 0.05). * *p* < 0.05, *** *p* < 0.001.

In vitality, baseline scores were higher in the control group (69.90 ± 20.54 vs. 57.88 ± 19.06), but post-intervention, the experimental group reported higher values (74.04 ± 15.78 vs. 66.73 ± 18.68). Significant effects were found for Group × Time (F(1,102) = 51.507, *p* < 0.001, η^2^ = 0.336) and Time (F(1,102) = 23.235, *p* < 0.001, η^2^ = 0.186). Post hoc comparisons indicated significant differences between groups at post-intervention (t(1,102) = −2.155, *p* = 0.034; d = 0.42), as well as pre–post improvements in the experimental group (t(51) = −9.817, *p* < 0.001; d = 0.47) (Figure 11).

**Figure 12:**
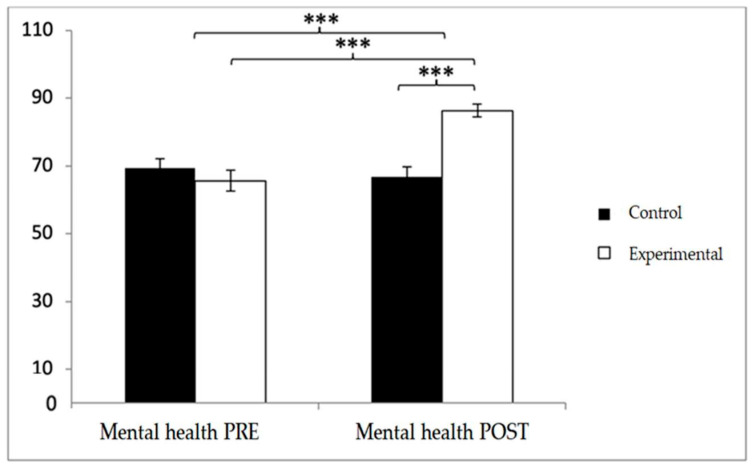
Inter- and intra-group comparisons for the mental health domain of the SF-12. Bars represent mean ± SD. Horizontal lines indicate significant differences between groups or time points (*p* < 0.05). *** *p* < 0.001.

In mental health, baseline scores were higher in the control group (69.38 ± 19.51 vs. 65.62 ± 22.69), but post-intervention values were greater in the experimental group (86.31 ± 13.91 vs. 66.75 ± 20.68). Significant effects were found for Group × Time (F(1,102) = 29.060, *p* < 0.001, η^2^ = 0.222), Time (F(1,102) = 17.414, *p* < 0.001, η^2^ = 0.146), and Group (F(1,102) = 6.296, *p* = 0.014, η^2^ = 0.058). Post hoc analyses revealed significant between-group differences at post-intervention (t(102) = −5.659, *p* < 0.001; d = 1.11) and significant pre–post improvements within the experimental group (t(51) = −9.817, *p* < 0.001; d = 1.10) (Figure 12).

**Figure 13:**
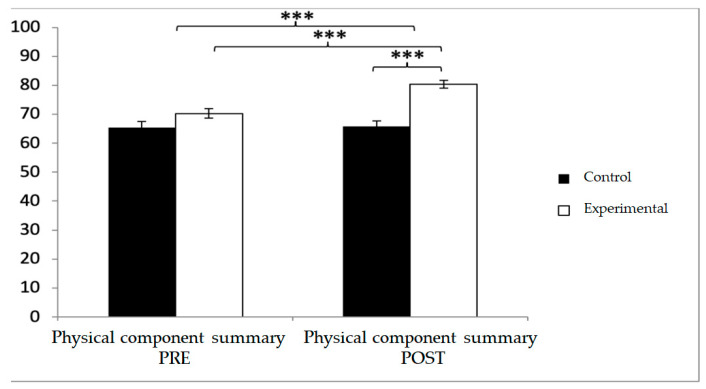
Inter- and intra-group comparisons for the physical component summary of the SF-12. Bars represent mean ± SD. Horizontal lines indicate significant differences between groups or time points (*p* < 0.05). *** *p* < 0.001.

For the physical component summary, baseline values were lower in the experimental group (70.27 ± 11.56 vs. 65.46 ± 14.28), but post-intervention values were significantly higher compared with the control group (80.39 ± 9.71 vs. 65.79 ± 13.15). Significant effects were found for Group × Time (F(1,102) = 23.005, *p* < 0.001, η^2^ = 0.184), Group (F(1,102) = 19.710, *p* = 0.023, η^2^ = 0.001), and Time (F(1,102) = 26.231, *p* < 0.001, η^2^ = 0.205). Post hoc analyses showed significant between-group differences at post-intervention (t(102) = −6.437, *p* < 0.001; d = 1.27) and significant within-group improvements in the experimental group (t(51) = −9.378, *p* < 0.001; d = 0.95) (Figure 13).

**Figure 14:**
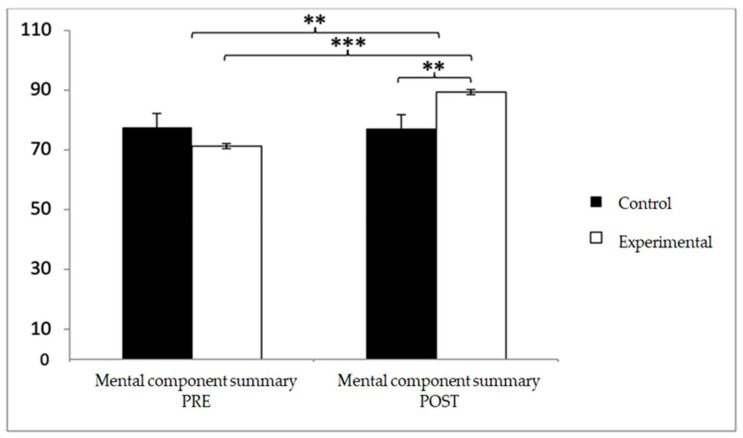
Inter- and intra-group comparisons for the mental component summary of the SF-12. Bars represent mean ± SD. Horizontal lines indicate significant differences between groups or time points (*p* < 0.05). ** *p* < 0.01, *** *p* < 0.001.

Finally, in the mental component summary, baseline scores were higher in the control group (77.56 ± 10.46 vs. 71.15 ± 11.73), but after the intervention, the experimental group reported superior outcomes (89.26 ± 6.06 vs. 77.15 ± 2.35). Significant effects were observed for Group × Time (F(1,102) = 13.938, *p* < 0.001, η^2^ = 0.120) and Time (F(1,102) = 12.748, *p* = 0.001, η^2^ = 0.111). Post hoc analyses confirmed significant differences between groups at post-intervention (t(102) = −2.652, *p* = 0.009; d = 0.52) and substantial pre–post improvements within the experimental group (t(51) = −12.715, *p* < 0.001; d = 1.93) (Figure 14).

## 4. Discussion

Findings from this randomized controlled trial indicate that participating in a 12-week structured Pilates program significantly enhances both nutritional status and HRQoL in older individuals with T2DM. These findings are particularly relevant given the complex health challenges faced by this population, who often experience multimorbidity, age-related physiological decline, and the psychosocial burden of managing a chronic disease [35,36,37].

With regard to nutritional status, our results showed that participants in the experimental group reported significantly higher post-intervention scores compared to those in the control group, with a medium effect size. This is an important contribution to the literature, as previous studies have predominantly focused on anthropometric outcomes—such as fat mass reduction or lean mass increase—rather than self-perceived nutritional well-being. For example, Fourie et al. [38] conducted an 8-week mat-based Pilates program in women aged over 60 years, consisting of three 60 min sessions per week, and observed significant reductions in body fat and improvements in lean body mass. Likewise, Carrasco-Poyatos et al. [39] implemented a 6-month Pilates intervention (two sessions per week, 60 min each) in postmenopausal women, reporting additional benefits for bone mineral density, a crucial determinant of functional independence in aging populations. While such studies underline the physiological adaptations elicited by regular Pilates training, our findings extend this evidence by demonstrating that Pilates may also improve subjective nutritional status, which integrates both physical perception and psychosocial awareness of health behaviors. The mechanisms by which Pilates could influence nutritional status are likely multifactorial. Regular physical activity has been shown to stimulate appetite regulation, promote healthier food choices, and reduce gastrointestinal discomfort, all of which can positively affect perceived nutritional well-being [19,35]. Moreover, Pilates emphasizes body awareness and mindfulness of movement, which may indirectly foster greater sensitivity to internal cues such as satiety and energy balance [40,41]. This enhanced interoceptive awareness, a key component of mind–body exercise, has been associated with improved eating behavior and reduced stress-related eating in older adults [40]. In addition, maintaining muscle mass and strength through Pilates may contribute to better metabolic control and appetite regulation, as muscle tissue plays a central role in glucose utilization and energy expenditure [42,43]. Bergamin et al. [42] conducted a 12-week mat-based Pilates program in postmenopausal women, consisting of two 60 min sessions per week, and reported significant improvements in lower-limb strength, postural control, and body composition, indicating enhanced muscle functionality and energy efficiency. Likewise, Tunar et al. [43] implemented an 8-week supervised Pilates intervention in adolescents with type 1 diabetes, performed three times per week for 60 min per session, and found significant improvements in both metabolic control and physical performance. These results support our findings, suggesting that regular, structured Pilates practice can improve muscle strength and metabolic efficiency, mechanisms that may explain the observed improvements in MNA scores. Consequently, Pilates could serve as a feasible adjunctive strategy for preventing malnutrition and supporting healthy aging in older adults with T2DM.

Interestingly, a subgroup analysis revealed that a small proportion of participants (≈11%) presented at risk of malnutrition at baseline (MNA = 17–23.5). Among these individuals, those allocated to the Pilates-based program exhibited an average increase of +2.1 points in MNA scores, while minimal changes were observed in the control group (+0.3 points). Although this analysis was exploratory and not powered for statistical inference, the positive trend suggests that mind–body exercise may be beneficial even in older adults at nutritional risk, possibly by enhancing appetite regulation, promoting social engagement, and improving self-perceived health. Further research with larger samples of undernourished participants is warranted to confirm these preliminary findings.

Equally relevant are the improvements observed in HRQoL, which encompasses multiple dimensions of health and well-being. Our data revealed significant post-intervention improvements in the experimental group across several domains of the SF-12, including general health, vitality, social functioning, and mental health, with moderate-to-large effect sizes. These findings are consistent with previous research indicating that exercise-based interventions exert broad psychosocial benefits in chronic disease populations.

For instance, Hartmann et al. [44] conducted an 8-week mindfulness-based stress reduction (MBSR) program in adults with type 2 diabetes mellitus, including weekly 2 h group sessions combined with daily home practice (≈45 min per day), and reported significant and sustained improvements in self-perceived health and psychological well-being. In contrast, Gabizon et al. [45] evaluated a 12-week Pilates training program in community-dwelling older adults (two 60 min sessions per week) and found no significant changes in self-reported health status, which the authors attributed to factors such as shorter program duration, lower adherence, and potential methodological limitations. Compared with these interventions, our 12-week Pilates-based exercise program, delivered twice weekly under supervision, not only improved physical and functional outcomes but also enhanced participants’ self-perceived health, suggesting that integrating mind–body awareness and controlled movement may provide additional psychological and behavioral benefits in older adults with type 2 diabetes.

The observed benefits of Pilates on mental health domains are particularly noteworthy. Participants in the experimental group reported large improvements in mental health scores, consistent with the notion that exercise acts as a protective factor against stress, anxiety, and depressive symptoms [46,47]. Unlike high-intensity exercise modalities, Pilates combines controlled breathing and mindful concentration with physical effort, potentially enhancing relaxation and emotional regulation. These effects are in line with previous evidence suggesting that mind–body practices such as yoga or tai chi contribute to improved emotional well-being, stress management, and self-esteem in individuals with T2DM. For instance, Nikkhah Ravari et al. [48] conducted a 12-week mindfulness-based stress reduction program in women with type 2 diabetes mellitus, involving weekly 90 min sessions combined with daily home meditation practice, and reported significant improvements in anxiety, depression, and glycemic control. Similarly, Dong et al. [49] performed a systematic review and network meta-analysis including more than 5.000 older adults and found that mind–body exercises, notably yoga, tai chi, and qigong, were associated with moderate to large reductions in anxiety and depressive symptoms. Collectively, these findings support the notion that Pilates, as a structured mind–body modality, may exert comparable psychosocial benefits through mechanisms involving relaxation, body awareness, and enhanced self-regulation [50]. Importantly, although glycemic control was not directly assessed in this study, previous evidence indicates that mental health and emotional well-being are strongly associated with better adherence to treatment and improved metabolic outcomes in people with T2DM [51]. Therefore, the psychosocial improvements observed here may indirectly contribute to more favorable diabetes self-management behaviors. Another key finding relates to vitality and social functioning, which improved significantly in the Pilates group compared to controls. Older adults with T2DM frequently report fatigue, social withdrawal, and reduced participation in community activities, factors that contribute to diminished HRQoL [52]. The group-based nature of Pilates may foster social connectedness, reduce isolation, and enhance motivation to engage in physical activity. Ruiz-Montero et al. [53] similarly reported improvements in daily functionality and vitality after a 24-week multicomponent training program that combined Pilates, aerobics, and health education in elderly Serbian women. The intervention consisted of three 60 min sessions per week, integrating mat-based Pilates movements, low-impact aerobics activities, and brief health-education segments on lifestyle and nutrition. Although their study excluded men and individuals with chronic diseases, such as diabetes, the findings demonstrated significant reductions in body fat mass and improvements in physical function and perceived vitality. Our results add novelty by showing that even a shorter 12-week Pilates-based program, performed twice weekly and tailored to older adults with type 2 diabetes, can yield comparable benefits in terms of functional capacity and perceived health in a more heterogeneous clinical population.

Regarding the physical role and physical functioning domains, no statistically significant improvements were observed following the Pilates intervention, and effect sizes were negligible. This lack of significant change may be related to the cumulative burden of T2DM-related complications such as neuropathy, sarcopenia, and impaired bone health, which can limit responsiveness to exercise-based interventions [54]. Nevertheless, maintaining functional performance without further decline is clinically meaningful in this population, as it contributes to preserving independence and reducing fall risk [55]. In this context, Pilates appears to be a feasible and safe option for stabilizing physical capacity and supporting overall mobility in older adults with limited exercise tolerance. Taken together, these results support the notion that Pilates can be considered a safe, adaptable, and effective adjunct to conventional care for older adults with T2DM. By targeting both physical and psychosocial domains, Pilates may help mitigate the multidimensional impact of diabetes and aging, thereby contributing to healthier aging trajectories. Still, several limitations should be acknowledged. The intervention lasted only 12 weeks, and it is unclear whether the benefits observed can be sustained over longer periods. The study sample was recruited from a single urban area, which may limit generalizability to other populations. Finally, Although this was a randomized controlled trial, participants’ motivation and prior interest in exercise may still have influenced adherence and perceived benefits, which should be considered when interpreting the findings.

Future research should address these limitations by conducting multicenter trials with longer follow-up, including diverse populations, and integrating multidisciplinary interventions combining exercise with dietary and psychosocial support. Such approaches may further optimize nutritional outcomes and HRQoL in older adults with T2DM.

## 5. Conclusions

This randomized controlled trial demonstrated that a 12-week Pilates-based exercise program significantly improved nutritional status and health-related quality of life in older adults with type 2 diabetes mellitus. The intervention produced benefits across both physical and psychosocial dimensions, with particularly notable improvements in general health, vitality, social functioning, and mental health. Importantly, the program was conducted under professional supervision, with exercises individually adapted to participants’ functional capacities, and no adverse events or exercise-related injuries were reported throughout the intervention, supporting the safety of Pilates in this population. These findings highlight Pilates as a safe, feasible, and effective adjunctive therapy that may contribute to the multidimensional management of diabetes and promote healthier aging. While the results are promising, future studies with larger and more diverse samples, longer follow-up periods, and combined interventions addressing diet, exercise, and psychosocial support are warranted to confirm the sustainability and broader applicability of these outcomes. Integrating Pilates into comprehensive diabetes care programs could represent an innovative strategy to enhance well-being, independence, and quality of life in this vulnerable population.

## Figures and Tables

**Table 1:** Sociodemographic and clinical characteristics of participants at baseline, presented for the total sample and stratified by group.

		Total (*n* = 104)	Experimental (*n* = 52)	Control (*n* = 52)	*p* Value
Age		70.57 ± 3.15	70.67 ± 3.21	70.46 ± 3.12	0.632
Sex	Male	31 (29.81)	15 (48.39)	16 (51.61)	0.672
	Female	73 (70.19)	37 (50.68)	36 (49.32)	
Years with the disease		12.63 ± 7.58	13.10 ± 8.41	12.15 ± 6.70	0.720
Comorbidities	High blood pressure	27 (26.0)	16 (59.30)	11 (40.70)	0.417
	Obesity	12 (11.50)	4 (33.30)	8 (66.70)	
	Dyslipidemia	35 (33.70)	17 (48.60)	18 (51.40)	
	Cataracts	24 (23.10)	13 (54.20)	11 (45.80)	
	Osteoporosis	6 (5.8)	2 (33.30)	4 (66.70)	
Family history of diabetes	Yes	49 (47.10)	23 (46.90)	26 (53.10)	0.409
	No	55 (52.90)	29 (52.70)	26 (47.30)	
Tobacco use	Yes	55 (52.9)	22 (40.0)	33 (60.0)	0.249
	No	49 (47.10)	30 (61.20)	19 (38.80)	
Alcohol use	Yes	58 (55.80)	25 (43.10)	33 (56.90)	0.056
	No	46 (44.20)	27 (58.70)	19 (41.30)	
Nutritional Status		25.51 ± 3.60	25.46 ± 3.50	25.56 ± 3.73	0.533
SF-12 General Health		59.95 ± 21.12	58.37 ± 22.49	61.54 ± 19.74	0.163
SF-12 Physical Functioning		71.30 ± 23.75	71.15 ± 24.02	71.44 ± 23.71	0.807
SF-12 Physical Role		69.71 ± 38.43	79.81 ± 28.46	59.62 ± 44.32	0.003
SF-12 Emotional Role		76.97 ± 25.75	73.46 ± 28.44	80.48 ± 22.47	0.014
SF-12 Social Functioning		89.06 ± 18.44	87.65 ± 19.69	90.46 ± 17.19	0.360
SF-12 Bodily Pain		70.50 ± 21.40	71.75 ± 19.93	69.25 ± 22.90	0.347
SF-12 Vitality		63.89 ± 20.62	57.88 ± 19.10	69.90 ± 20.54	0.858
SF-12 Mental Health		67.50 ± 21.14	65.62 ± 22.68	69.38 ± 19.51	0.073
SF-12 PCS		67.87 ± 13.15	70.27 ± 11.56	65.46 ± 14.27	0.118
SF-12 MCS		74.36 ± 115.52	71.15 ± 11.73	77.56 ± 10.46	0.536

Data are expressed as mean ± standard deviation (SD) for continuous variables and as absolute frequencies and percentages (*n* (%)) for categorical variables. Values in parentheses represent the percentage of participants within each group. SF-12: (Medical Outcome Study Short Form-12); PCS: Physical Component Summary; MCS: Mental Component Summary.

## Data Availability

The datasets generated and analyzed during the current study are available from the corresponding author upon reasonable request. Due to the sensitive nature of the information collected, participants were assured that their data would remain confidential and would not be publicly disclosed.

## Abbreviations

The following abbreviations are used in this manuscript:

T2DM	Type 2 Diabetes Mellitus
HRQoL	Health-Related Quality of Life
MNA	Mini Nutritional Assessment
SF-12	12-Item Short Form Health Survey
PCS	Physical Component Summary
MCS	Mental Component Summary
ANOVA	Analysis of Variance
SD	Standard Deviation
CG	Control Group
EG	Experimental Group
